# Enhancing the Immune Response of a Nicotine Vaccine with Synthetic Small “Non-Natural” Peptides

**DOI:** 10.3390/molecules25061290

**Published:** 2020-03-12

**Authors:** Hoang-Thanh Le, Nya L. Fraleigh, Jordan D. Lewicky, Justin Boudreau, Paul Dolinar, Nitin Bhardwaj, Francisco Diaz-Mitoma, Sabine Montaut, Sarah Fallahi, Alexandrine L. Martel

**Affiliations:** 1 Health Sciences North Research Institute, 56 Walford Road, Sudbury, ON P3E 2H3, Canada; nfraleigh@hsnri.ca (N.L.F.); jlewicky@hsnri.ca (J.D.L.); jboudreau@hsnri.ca (J.B.); dolinar.paul@gmail.com (P.D.); Sara.Fallahi@turnstonebio.com (S.F.); amartel@hsnri.ca (A.L.M.); 2 Department of Biology, Laurentian University, 935 Ramsey Lake Road, Sudbury, ON P3E 2C6, Canada; 3 Department of Chemistry and Biochemistry, Biomolecular Sciences Programme, Laurentian University, 935 Ramsey Lake Road, Sudbury, ON P3E 2C6, Canada; smontaut@laurentian.ca; 4 Northern Ontario School of Medicine, 935 Ramsey Lake Road, Sudbury, ON P3E 2C6, Canada; mitoma99@gmail.com; 5 Division of Comparative Medicine, University of Toronto, 1 King’s College Circle, Toronto, ON M5S 1A8, Canada; nitin.bhardwaj@utoronto.ca; 6 VBI Vaccines Inc., 310 Hunt Club Road East, Suite 201, Ottaway, ON K1V 1C1, Canada

**Keywords:** peptide solid phase synthesis, non-natural peptides, vaccine delivery, IL-1β, bacterial derived adjuvant, phagocytic cells, dendritic cells, macrophages, immune responses

## Abstract

The addictive nature of nicotine is likely the most significant reason for the continued prevalence of tobacco smoking despite the widespread reports of its negative health effects. Nicotine vaccines are an alternative to the currently available smoking cessation treatments, which have limited efficacy. However, the nicotine hapten is non-immunogenic, and successful vaccine formulations to treat nicotine addiction require both effective adjuvants and delivery systems. The immunomodulatory properties of short, non-natural peptide sequences not found in human systems and their ability to improve vaccine efficacy continue to be reported. The aim of this study was to determine if small “non-natural peptides,” as part of a conjugate nicotine vaccine, could improve immune responses. Four peptides were synthesized via solid phase methodology, purified, and characterized. *Ex vivo* plasma stability studies using RP-HPLC confirmed that the peptides were not subject to proteolytic degradation. The peptides were formulated into conjugate nicotine vaccine candidates along with a bacterial derived adjuvant vaccine delivery system and chitosan as a stabilizing compound. Formulations were tested *in vitro* in a dendritic cell line to determine the combination that would elicit the greatest 1L-1β response using ELISAs. Three of the peptides were able to enhance the cytokine response above that induced by the adjuvant delivery system alone. *In vivo* vaccination studies in BALB/c mice demonstrated that the best immune response, as measured by nicotine-specific antibody levels, was elicited from the conjugate vaccine structure, which included the peptide, as well as the other components. Isotype analyses highlighted that the peptide was able to shift immune response toward being more humorally dominant. Overall, the results have implications for the use of non-natural peptides as adjuvants not only for the development of a nicotine vaccine but also for use with other addictive substances and conventional vaccination targets as well.

## 1. Introduction

Peptides have been used in various applications in medicine, immunology, and pharmacology for the creation of substances including macrocyclic antibiotics, integrin inhibitors, anticancer agents, neuromodulators, opioids, and hormones [1,2,3,4,5,6]. A new direction in peptide research involves the use of synthetic peptides in immunotherapies and vaccines where they can act as both antigens and as components of adjuvants and delivery systems [7,8,9,10]. A number of peptide vaccines are in various stages of development, offering hope for the control of numerous diseases caused by HIV, Influenza A, and Hepatitis C [11,12,13].

The frequency of different lengths of peptide sequences in the universal proteome database has been investigated using bioinformatics tools. Interestingly, while all possible four amino acid (aa) sequences occur at least once in humans and all other organisms, there are certainly 5 aa or 6 aa combinations that are absent [14,15]. Short peptide sequences of 5–6 aa have been shown to be important in a variety of biological processes, including immune activation, and combining vaccine candidates with immunomodulatory peptides has been an effective strategy to enhance immunogenicity [16,17,18,19]. Kobinger’s group screened the UniRef100 universal proteome database and identified a large pool of rare or “non-natural” 5 aa pentapeptides that were screened for immunomodulatory activity [20,21]. Many of the pentapeptides were able to strengthen immune responses when added to a vaccine complex, without increasing the possibility of adverse inflammatory reactions. The pentapeptide (Peptide **1**, Table 1) demonstrated superior activity in combination with both influenza and hepatitis B vaccines [22]. Overall, there are several advantages for rare short peptides as immunostimulants. They can improve both humoral and cellular immune responses to vaccine antigens while remaining non-immunogenic themselves. In addition, they can also work synergistically with other adjuvants in improving immune responses while being both non-toxic and easy/inexpensive to prepare [21].

The conjugation of a non-natural pentapeptide with a hapten antigen such as nicotine may provide the necessary signals to enable the recognition and stimulation of an immune response. Tobacco smoking is a global pandemic causing 8 million deaths per year [23] and yet smoking cessation remains relatively unsuccessful due to nicotine addiction. The development of a therapeutic nicotine vaccine offers one of the most promising treatment options to date, where the induction of a potent and selective antibody response against nicotine would block its addictive effects in the central nervous system. As nicotine is non-immunogenic, immunogenicity needs to be increased through conjugation to a peptide or protein carrier, and the use of adjuvants [24]. A successful vaccination strategy is, therefore, dependent upon the appropriate hapten design, carrier protein, and adjuvant, all of which affect both the magnitude and affinity of the immune response elicited [25,26,27]. We have been developing a mucosal nicotine vaccine that employs a naturally adjuvanted delivery system derived from the outer membrane of bacteria (*Neisseria meningitidis* or *Vibrio cholerae*) [28,29,30], and a synthetic non-natural peptide that facilitates nicotine conjugation. The preparation of the adjuvant system, which we have coined bacterial derived adjuvant (BDA), was previously described by our group [28,29]. Importantly, our nicotine vaccine is unlike previous vaccines [31,32,33,34,35] and has been designed to be administered via an intranasal (IN) route. The BDA vaccine delivery system generates a robust immune response by stimulating IL-1β production through Toll-like receptor 4 (TLR4), a potent mechanism for mucosal immunity [28], resulting in high mucosal and systemic antibody levels in mice [28,29]. In this report, we further studied the potential use of the non-natural pentapeptides for enhancing the immune response of our nicotine vaccine. We report the synthesis and evaluation of several non-natural peptides (Table 1) as potential immunostimulants. Nicotine vaccines were formulated in the presence of the peptides and the BDA at a 1:5 ratio of BDA:Peptide (*w*/*w*, based on BDA protein). At this ratio, the degree of nicotine conjugation was up to five times higher than without the peptides (as visualized by TLC with detection by Dragendorff reagent). The formulations were first screened *in vitro* for their ability to induce IL-1β production. The best formulation was carried forward for *in vivo* studies in mice, where the contribution of each of the components of the vaccine to the immune response was assessed by measuring the levels of both nicotine-specific IgG and different IgG isotypes. Overall, the results clearly indicate that the non-natural peptides are capable of enhancing the immune response toward our nicotine vaccine.

## 2. Results and Discussion

**Figure 1:**
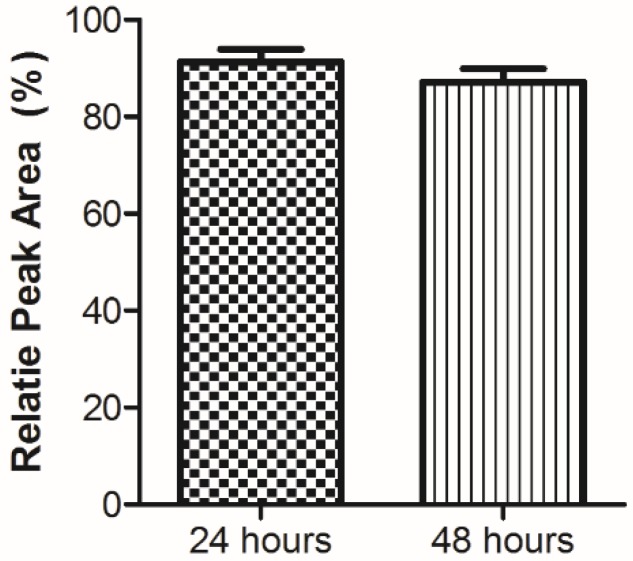
Peptide stability in plasma. Pentapeptide **1** was incubated in human plasma at 37 °C. Levels of the peptide remaining after various time points were analyzed by RP-HPLC in the presence of 0.1% trifluoroacetic acid and detected by absorbance at 210 nm. Peptide amounts were calculated relative to quantities determined at time point zero, and data shown are the average ± SEM of three separate experiments. No statistical difference was found between the two time points.

The peptides that were prepared included the pentapeptide previously reported by Kobinger’s group [20,21,22], as well as several extended analogs that contain this 5 aa sequence (Table 1). Peptides **2** and **3** are dodecapeptides that have additional lysine and glutamic acid residues, respectively, to increase branching points, as well as phenylalanine residues to promote pi–pi intermolecular interactions, all of which provide additional sites for nicotine conjugation and could aid in nanoparticle self-assembly [36]. Peptide **4** contains a succinamic acid residue at the N-terminal for the potential binding to nicotine. All of the peptides were synthesized using our standard solid-phase synthesis protocol with some modifications [5] and were obtained in moderate-to-good yield with high purity (Table 2). The plasma stability of pentapeptide **1** was assessed using RP-HPLC and our previously published methods [37], with results confirming that the peptide sequence was not recognized and cleaved by proteolytic enzymes (Figure 1), and would likely improve the overall stability of the conjugate vaccine *in vivo*.

**Figure 2:**
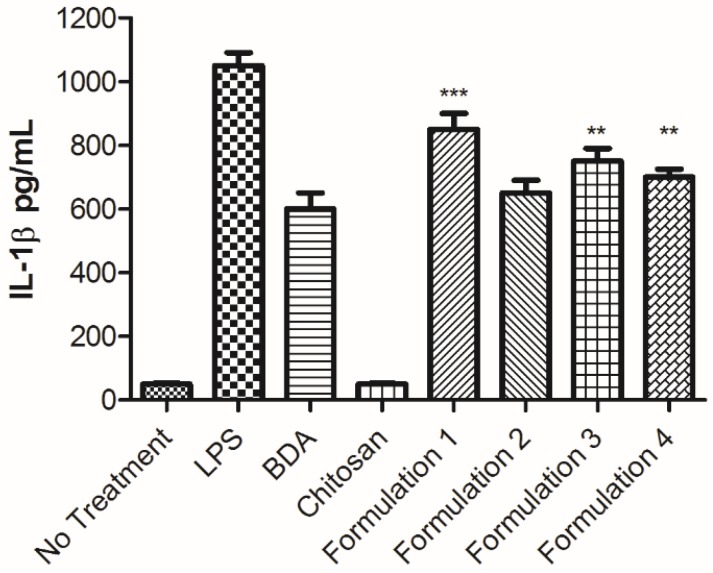
Levels of IL-1β produced by JAWSII after 48 h. JAWSII, immortalized bone-marrow-derived dendritic cells, were seeded at a concentration of 10^6^ cells/mL/well in a 12-well plate and left untreated or treated with either 1 μg/mL LPS from *E. coli* 0111:B4, vaccine components (bacterial derived adjuvant (BDA): 1 μg/ mL, chitosan: 10 μg/mL), or the different peptides (1–4) containing formulations (1 μg/mL based on BDA). Supernatants were collected from the cells after 48 h of treatment and levels of IL-1β were analyzed via ELISA. N = 6 ± SEM. Statistical significance was determined by an ANOVA with a Tukey HSD. *** *p* < 0.001 and ** *p* < 0.01 as compared to BDA.

Dendritic cells are a crucial link between the innate and acquired immunity as they are adept in phagocytosis, secretion of cytokines, and antigen presentation. Activated antigen-presenting cells are required to initiate T-cell-mediated immune responses to a foreign substance [38]. By using IL-1β secretion to detect the activation of these professional antigen-presenting cells, the initial response was quickly assessed. Treatment with the synthetic peptides alone does not induce IL-1β release when evaluated by ELISA (data not shown). This was not surprising, as functional IL-1β secretion is dependent upon two signals, resulting in the activation of the inflammasome and caspase 1 to cleave the pro-IL-1β [39]. When combined with the BDA, in respective nicotine vaccine formulations, IL-1β production was maintained with all of the peptides (Figure 2). Interestingly, formulation with peptides **3** or **4** and their extended amino acid sequences, both enhanced the cytokine response to approximately the same extent, while there was no statistical difference with the formulation containing the extended peptide **2** as compared to the BDA alone. Cytokine levels with the formulation containing the core pentapeptide **1** were enhanced to the greatest degree. This stronger proinflammatory cytokine release would theoretically drive a stronger adaptive immune response against nicotine, resulting in a more effective vaccine. Overall, each of the peptides can be used with BDA to improve the degree of nicotine conjugation, while some offer the additional benefit of increased immunostimulatory activity. In this study, the formulation containing peptide **1** was selected to be used for *in vivo* evaluations.

**Figure 3:**
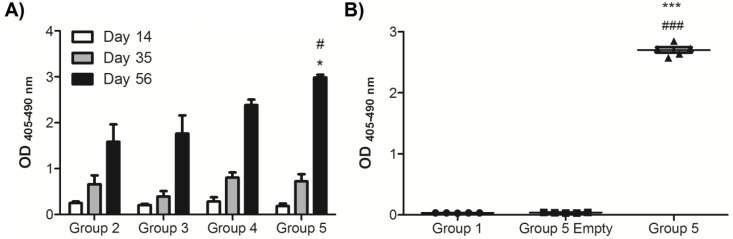
Levels of anti-nicotine IgG. Female BALB/c mice were administered intranasal installations of either PBS (Group 1) or different nicotine vaccine formulations: [BDA/Nic Hapten] (Group 2), [BDA/Nic Hapten /Chitosan] (Group 3), [BDA/Peptide **1**/Nic Hapten] (Group 4), and [BDA/Peptide **1**/Nic Hapten/Chitosan] (Group 5); Group 5 Empty refers to the delivery system without nicotine. Blood was collected two weeks post-vaccination (Day 14, 35, 56, and 77). (**A**) Anti-nicotine IgG from individual sera and diluted 1:300. Data are represented as ± SEM (n = 4–5 for each group). Statistical significance was determined by a Kruskal–Wallis with a Dunn’s multiple comparison test * *p* < 0.05 as compared to Group 2 and # *p* < 0.05 as compared to Group 3. (**B**) In a repeated trial, the total anti-nicotine IgG present was detected in sera (1:600 dilution). Each point represents an individual mouse and data are represented as ± SEM, *n* = 5. Statistical significance was determined by an ANOVA with a Tukey HSD. *** *p* ≤ 0.001 as compared to Group 1 and ### *p* ≤ 0.001 as compared to Group 5 Empty.

We evaluated the contribution of each of the components of the IN delivered nicotine vaccine to the immune responses *in vivo* using BALB/c mice (Figure 3), including pentapeptide **1** (Table 1). The formulation containing only the BDA induced the lowest levels of nicotine-specific IgG. Overall, the strongest immune responses were observed in the formulations containing the synthetic pentapeptide **1**, and responses were significantly improved as compared to Groups 2 and 3, which lack the pentapeptide. Peptide **1** also appears to reduce the variability between the individual mice vaccinated IN, as seen in Figure 3A, and including chitosan as a stabilizing component served to increase the immune responses, which was most evident after the third blood collection. This is in line with our previous publication, which demonstrated that the IN conjugate-nicotine vaccine was able to prevent nicotine from entering the brain after vaccination using [^3^H]-nicotine challenges *in vivo*, leading to a fourfold reduction in brain nicotine concentrations, as compared to controls [28].

**Figure 4:**
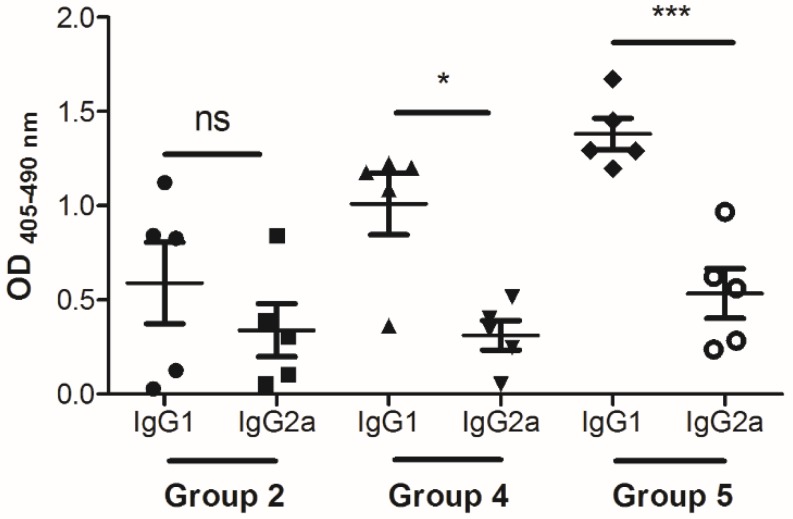
Levels of anti-nicotine IgG isotypes. Female BALB/c mice were administered intranasal instillations of different nicotine vaccine formulations and blood was collected two weeks post-vaccination. Individual mouse sera were diluted and anti-nicotine IgG1 and IgG2a were assessed on day 77. Data are represented as ± SEM, n = 5. Statistical significance was determined for Group 5 by an unpaired 2-tailed T-test, *** *p* = 0.0006, and for Group 4 by a Mann–Whitney test, * *p* = 0.0317.

The full vaccine formulation containing the pentapeptide, chitosan, and BDA provided the best results, with antibody levels that, upon repeated trials, were consistently saturated by the fourth bleed, even at a dilution of 1:600 (Figure 3B). In subsequent investigations, we were able to determine that dilutions on the order of greater than 10^4^ were needed in order to stop saturation (data not shown). In order to conclude that the delivery system was not inducing non-specific reactions during the ELISA protocol, an additional negative control was added in the repeated trial (Group 5 Empty). Peptide **1** also appears to shift the anti-nicotine IgG isotype response toward a more IgG1-dominant phenotype, suggesting a stronger polarization toward a Th2 response, as seen in Figure 4. The BDA/Nic Hapten formulation had a balanced IgG1:IgG2a ratio, and when Peptide **1** was added in Groups 4 and 5, there is a significant shift toward an IgG1-dominated response, which is strongest in the full formulation (Group 5).

It appears that the *in vitro* assessment of IL-1β is a useful tool to screen these adjuvant formulations for potential *in vivo* performance as it can enhance antigen presentation and humoral immune responses [40,41,42,43,44]. This is reflected in the data as the BDA group alone was not as effective as an adjuvant of the nicotine vaccine as compared to Formulation 1 and vaccine Group 5 for *in vitro* and *in vivo* testing, respectively.

## 3. Materials and Methods

### 3.1. Peptide Synthesis

Peptides were synthesized by solid phase synthesis on a Symphony 12-Channel Multiplex Synthesizer (Protein technologies Inc., Tucson, AZ, USA), using a combination of manual and automated synthesis, following the conventional Fmoc/HBTU methodology. As an example for peptide **1**, the Fmoc-Cys(Trt)-NovaSynTGT Resin (ca. 500 mg; 0.2 meq/g; 1 mmol) was swollen in dimethylformamide (DMF) and then thoroughly washed using DMF (2 × 5 mL). The remaining amino acids were coupled on the resin by repeated cycles of Fmoc-deprotection, activation, and coupling. Each deprotection step involved a double treatment with 20% piperidine in DMF (20 min total) followed by washing with DMF (6 × 5 mL) and ninhydrin testing. Each activation step was performed by mixing the protected amino acid (0.8 mmol in 2.7 mL DMF) with HBTU (0.7 mmol in DMF) and a slight excess of *N*,*N*-diisopropylethylamine (1.6 mmol) for 5 min. The activated amino acid was then allowed to couple to the resin for 1.5 h with nitrogen bubbling. The peptide-resin was thoroughly washed with DMF (6 × 5 mL) after coupling and a ninhydrin test was performed to ensure completion. Upon completion of synthesis, the N-terminal Fmoc group was removed under standard conditions, and the peptide-resin was washed with DMF.

Peptides were cleaved using a trifluoroacetic acid (TFA) cocktail (TFA: Thioanisole: Phenol: H_2_O: 1,2-ethanedithiol: Triisopropylsilane (81.5:5:5:2.5:1)) for 3 h. The products were then collected and washed with 2 × 5 mL TFA. The filtrate was evaporated *in vacuo* and then precipitated by using cold ether (30 mL). After decanting the ether layer, the peptide was washed with cold ether (2 × 10 mL), dried in a desiccator overnight, dissolved in double-distilled H_2_O, and lyophilized to yield the crude peptide.

Crude peptides were purified by HPLC on a Dionex UltiMate 3000 UHPLC system (Thermo Scientific, Mississauga, ON, Canada) fitted with a PrepPak Cartridge (C-18, 15–20 µm, 25 mm × 100 mm, Waters, Mississauge, ON, Canada) on a PrepLC 25 Module (Waters, Mississauga, ON, Canada) using a binary gradient of aqueous 0.1% TFA (solvent A) and 80% acetonitrile containing 0.1% TFA (solvent B) at a flow rate of 10.0 mL/min. The gradient consisted of 10 min of solvent A running through the column. The gradient then began to transition from solvent A to solvent B over 30 min. Solvent B was then run through the column for an additional 20 min. The eluent was monitored at 210 and 260 nm and collected into fractions. UV absorbing fractions were collected and then characterized by analytical HPLC and electrospray ionization mass spectrometry (ESI-MS, Shimadzu Corporation, Kyoto, Japan). Analytical HPLC was carried out on a Shimazdu modular HPLC system fitted with a reverse-phase column (Restek Ultra C-18, 3µ m, 4.6 mm × 150 mm) and running solvent A (0.1% aqueous TFA) and solvent B (0.1% TFA in 95% acetonitrile) at 0.7 mL/min. The first 10 min consisted solely of solvent A running through the column. The gradient then began to transition from solvent A to solvent B over 20 min. Solvent B was then run through the column for an additional 10 min. The purified peptide was lyophilized and analyzed by ESI-MS on an 6120 Quadropole LC/MS (Agilent Technologies, Inc., Toronto, ON, Canada) operating in positive mode with a scanning range from 105 to 1300 *m*/*z*, a N_2_ gas temperature of 350 °C, and a voltage at 4 kV. Fractions of sufficient purity were combined and lyophilized to yield the desired peptides at a high level of purity. The detailed characterization and identification of peptides are summarized in Supplementary Materials document.

### 3.2. Plasma Stability Analysis

Blood was collected from healthy volunteers in EDTA blood collection tubes (BD Vacutainer, Mississauga, ON, Canada). Plasma was collected after centrifugation at 900× *g* and 20 °C for 10 min with decreased deceleration, aliquoted, and stored at −80 °C. All subjects gave their informed consent for inclusion before they participated in the study. The study was conducted in accordance with the Declaration of Helsinki, with all protocols approved by the Research Ethics Board at Health Sciences North Research Institute (Protocol # 18-061).

The stability of pentapeptide **1** in human plasma was analyzed using previously published methods [37].

### 3.3. Formulation and Vaccine Preparation

Formulations (1–4) were prepared by conjugation of the corresponding peptide (**1**–**4**,**Table 1**) with the BDA [28,29,30] (2–5 mg/mL in water based on protein content determined using Thermo Scientific Pierce BCA Protein Assay Kit) at pH 5–6 in the presence of EDC coupling reagent (Sigma Aldrich, Saint Louis, MO, USA, 10 equiv. based on BDA protein content).

Vaccines (Table 3) were prepared as previously described [29] by conjugation of 3′-aminomethylnicotine (Toronto Research Chemicals Inc., Toronto, ON, Canada, 25 mg/mL in MeOH) with the BDA component (2–5 mg/mL in water based on protein), with or without peptide **1** and the addition of chitosan as a stabilizing compound at pH 5–6 in the presence of EDC coupling reagent (10 equiv. based on nicotine hapten concentration). Nicotine was quantified during the conjugation reaction using the UV absorption of derived nicotine as a standard at 265 nm and detected by TLC on Silica gel 60 F254 (EM Science, Gibbstown, NJ, USA) with Dragendorff reagent staining.

All final conjugation products were purified by dialysis in HEPES buffer with 0.01% Tween 80, and vaccines were lyophilized using different freeze-drying techniques. Particle size as a parameter for stability analysis was analyzed by dynamic light scattering with a Zetasizer NanoZS (Malvern Instruments, Malvern, United Kingdom) equipped with a 4 mW 633 nm He–Ne laser, an avalanche photodiode positioned 175° to the beam, and a temperature-controlled cuvette holder. Instrument parameters were determined automatically along with measurement times. The conjugate vaccine systems (2–5 mg/mL based on nicotine concentration) were stored at either 4 °C (solution form) or at room temperature (solid form) before being used for *in vivo* animal studies or *in vitro* testing.

### 3.4. In Vitro Studies

To investigate the peptides’ effect on nicotine vaccine formulations *in vitro*, JAWSII cells (bone marrow-derived dendritic cell line from p53 -/- C57BL/6 mice) were grown to confluency in RPMI 1640 supplemented with 8% FBS (Gibco, Life Technologies, Mississauga, ON, Canada), 1% penicillin/streptomycin, and 5 ng/mL GM-CSF (Gibco, Life Technologies, Mississauga, ON, Canada) in the presence of 5% CO_2_ at 37 °C. Cells were seeded at a concentration of 10^6^ cells/mL and treated with either 1 µg/mL LPS from *E. coli* 0111:B4 (Sigma, St. Louis, MO, USA), vaccine components BDA (1 µg/mL) or chitosan (10 μg/mL), or formulations 1–4 (1 µg/mL based on BDA) for 48 h. Supernatants were analyzed for levels of IL-1β using a commercial ELISA (eBioscience, San Diego, CA, USA). The assay was performed as per the instructions from the manufacturer, and the limit of detection was 7.8 pg/mL.

### 3.5. In Vivo Vaccination

Six- to eight-week-old female BALB/c mice were purchased from Charles River (Montreal, QC, Canada). Mice were housed at the Laurentian University Animal Care Facility and were supplied food and water *ad libitum*. All protocols were approved by the Animal Care Committee at Laurentian University (Protocol #2013-04-02). The animal room was maintained at a temperature of 21 ± 2 °C and a relative humidity of 55% ± 5%. These parameters were recorded daily in addition to maintaining 12 h light and dark cycles. Mice were allowed to acclimatize to their surroundings for one week prior to the commencement of the experimental protocol and were randomly placed into groups of 5. Animals were anesthetized using either a ketamine/xylazine (Wyeth, Guelph, ON, Canada/ LLOYD Inc., Shenandoah, IA, USA) cocktail or isoflurane.

All 5 groups of animals were immunized IN with the prime vaccination on day 0, and booster vaccinations on days 21, 42, and 63 with a fixed amount of vaccine corresponding to 10 µg of nicotine (20 µL total volume, 10 µL per nare). Group 1 received PBS, while Groups 2–5 received vaccines 2–5, respectively. Sera were harvested from blood samples collected via retro-orbital bleed or the submandibular vein on days 14, 35, 56, and 77. Nicotine-specific antibodies in sera were assayed following a previously reported ELISA protocol [28,29].

## 4. Conclusions

The immunomodulatory mechanisms of certain non-natural short peptide sequences continue to be reported. We have shown here that the immunostimulatory activity of a pentapeptide member of this family is capable of enhancing antigen-specific immune responses toward our nicotine vaccine candidate. Overall, the peptide was able to increase nicotine conjugation with the BDA, with the stability of the conjugate system likely also improved. Importantly, the peptide improved immune response homogeneity, and shifted antibody isotypes toward a predominantly IgG1 response. The results have implications for the use of non-natural peptides as adjuvants in the development of new vaccines against not only conventional targets such as bacteria and viruses, but against other addictive substances as well.

## Figures and Tables

**Table 1:** Synthetic peptides and amino acid sequences.

Peptide	Amino Acid Sequence
**1**	**KWCEC**
**2**	KWCECKFFKFFG
**3**	KWCECEFFEFFG
**4**	Succinamic-KWCEC

**Table 2:** Synthetic peptide identification and characterization.

Peptide	Ninhydrin Test	Scale and Yield	HPLC t_R_ (min)	Mass Spectrometry	
**1**	Positive	300 nmol 100 mg (50.0%)	14.8	Calculated: Measured:	667.25 668.1 (M + H)^+^
**2**	Positive	200 nmol 240 mg (76.5%)	20.8	Calculated: Measured:	1568.73 785.5 (M + 2H)^2+^
**3**	Positive	200 nmol 45 mg (14.3%)	23.7	Calculated: Measured:	1571.8 786.4 (M + 2H)^2+^
**4**	Positive	100 nmol 37 mg (48.2%)	16.5	Calculated: Measured:	766.87 767.3 (M + H)^+^

**Table 3:** Vaccine group composition.

Vaccine	Composition
2	BDA + Nicotine
3	BDA + Nicotine/Chitosan
4	BDA + Nicotine + Peptide **1**
5	BDA + Nicotine + Peptide **1**/Chitosan

## Supplementary Materials

The following are available online. Figure S1: KWCEC peptide HPLC results (99.38% purity), Figure S2: KWCEC peptide ESI-MS results, Figure S3: KWCECKFFKFFG peptide HPLC results (97.32% purity), Figure S4: KWCECKFFKFFG peptide ESI-MS results. Figure S5: KWCECEFFEFFG peptide HPLC results (89.37% purity), Figure S6: KWCECEFFEFFG peptide ESI-MS results, Figure S7: Succinamic KWCEC peptide HPLC results (98.76% purity), Figure S8: Succinamic KWCEC peptide ESI-MS results.
