# Supplementary material for: Enhancing the Immune Response of a Nicotine Vaccine with Synthetic Small “Non-Natural” Peptides
Source: Molecules. 2020 Mar 12;25(6):1290. doi: 10.3390/molecules25061290 (PMC7143940; doi:10.3390/molecules25061290)
Supplement: Supplementary file 1 [file molecules-25-01290-s001.pdf]

APPENDIX A

Enhancing the immune response of a nicotine vaccine with synthetic small "non-natural" peptides.

Hoang-Thanh Le, Nya L. Fraleigh, Jordan D. Lewicky, Justin Boudreau, Paul Dolinar, Nitin Bhardwaj, Francisco Diaz-Mitoma, Sabine Montaut, Sarah Fallahi, and Alexandrine L. Martel.

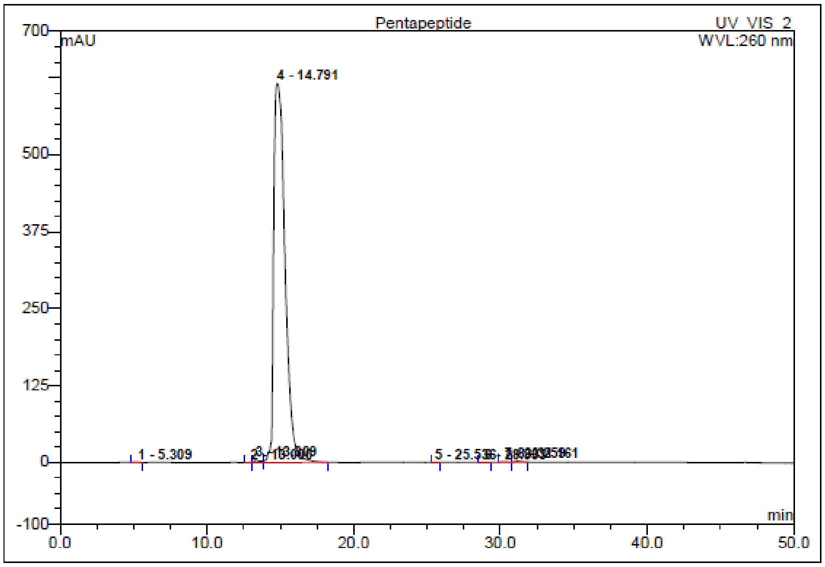

No.	Ret.Time min	Peak Name	Height mAU	Area mAU*min	Rel.Area %	Amount	Type
1	5.31	n.a.	0.651	0.223	0.04	n.a.	BMB
2	13.00	n.a.	0.674	0.184	0.03	n.a.	BM
3	13.31	n.a.	4.159	2.003	0.36	n.a.	M
4	14.79	n.a.	615.953	556.481	99.38	n.a.	MB
5	25.54	n.a.	0.197	0.070	0.01	n.a.	BMB
6	28.93	n.a.	0.406	0.150	0.03	n.a.	BMB
7	30.26	n.a.	0.608	0.258	0.05	n.a.	BM
8	31.16	n.a.	1.382	0.597	0.11	n.a.	MB
Total:			624.032	559.965	100.00	0.000	

Figure S1. KWCEC peptide HPLC results (99.38% purity).

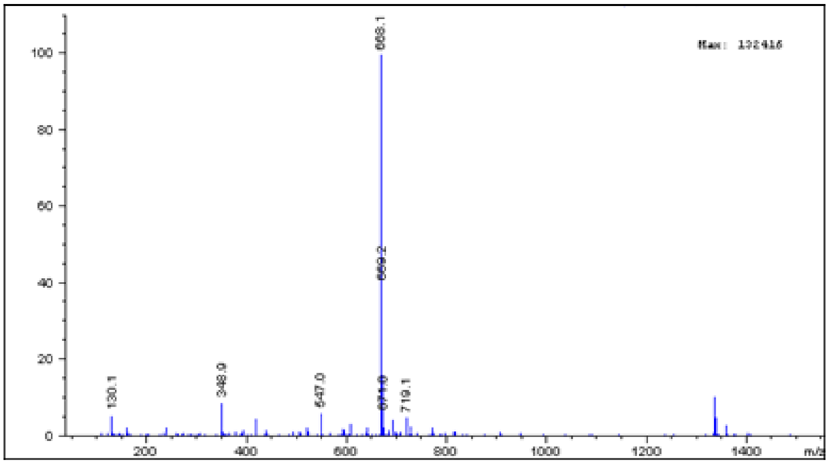

Figure S2. KWCEC peptide ESI-MS results.

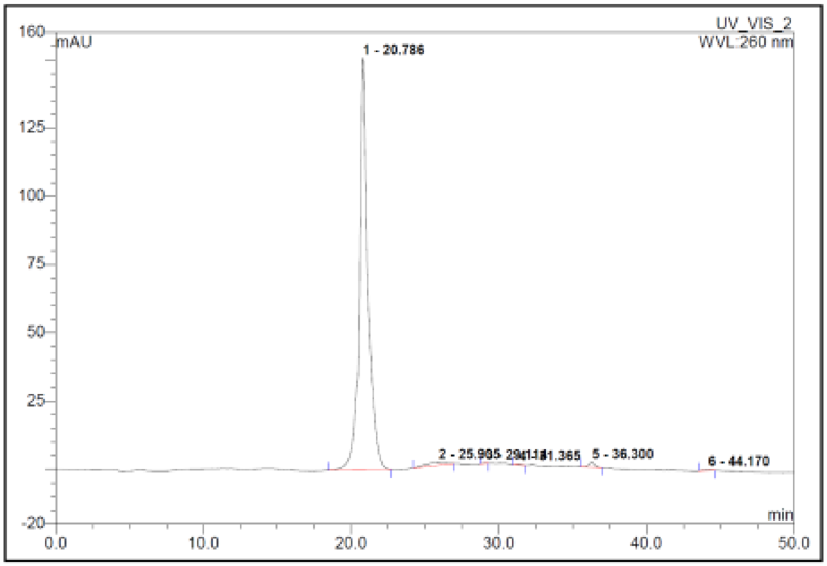

No.	Ret.Time min	Peak Name	Height mAU	Area mAU*min	Rel.Area %	Amount	Type
1	20.79	n.a.	150.743	102.805	97.32	n.a.	BMB
2	25.90	n.a.	0.912	1.601	1.52	n.a.	BMB
3	29.11	n.a.	0.142	0.052	0.05	n.a.	BMB
4	31.37	n.a.	0.378	0.132	0.12	n.a.	BMB
5	36.30	n.a.	1.788	0.848	0.80	n.a.	BMB
6	44.17	n.a.	0.351	0.200	0.19	n.a.	BMB
Total:			154.312	105.639	100.00	0.000	

Figure S3. KWCECKFFKFFG peptide HPLC results (97.32% purity).

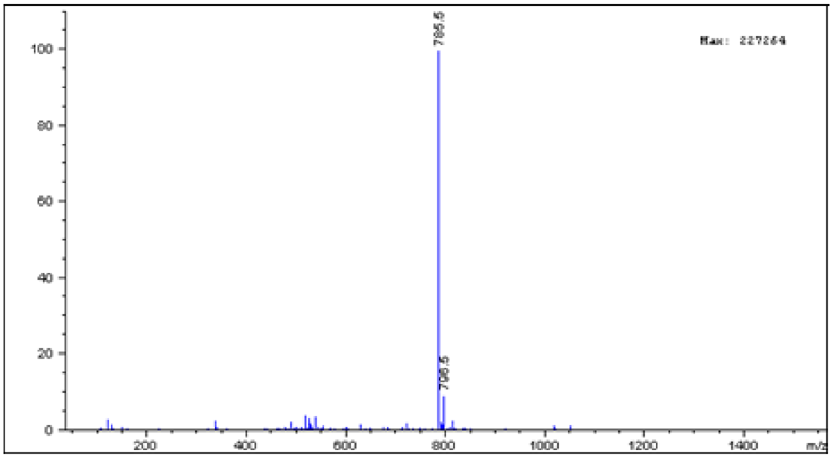

Figure S4. KWCECKFFKFFG peptide ESI-MS results.

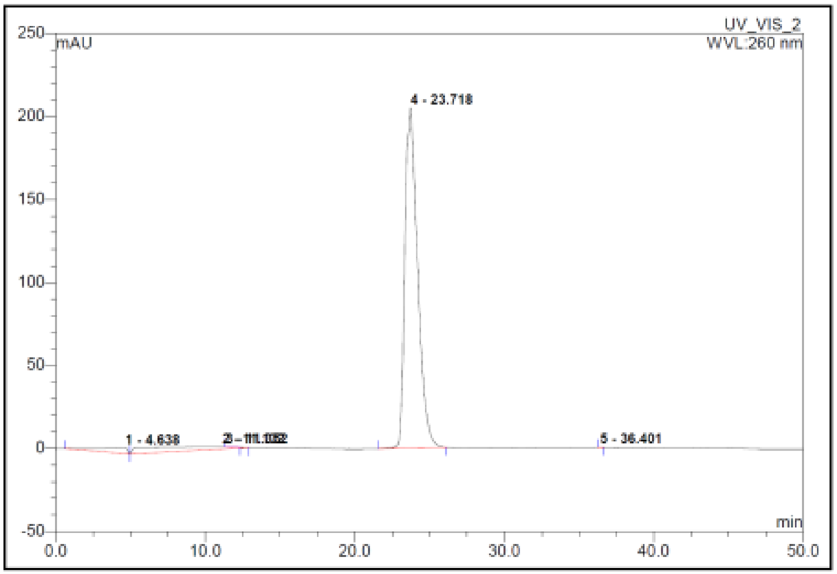

No.	Ret.Time min	Peak Name	Height mAU	Area mAU*min	Rel.Area %	Amount	Type
1	4.64	n.a.	3.347	6.939	3.06	n.a.	BMB
2	11.13	n.a.	1.374	17.067	7.52	n.a.	BMB
3	11.35	n.a.	0.052	0.084	0.04	n.a.	Rd
4	23.72	n.a.	205.059	202.815	89.37	n.a.	BMB
5	36.40	n.a.	0.122	0.024	0.01	n.a.	BMB
Total:			209.954	226.929	100.00	0.000	

Figure S5. KWCECEFFEFFG peptide HPLC results (89.37% purity).

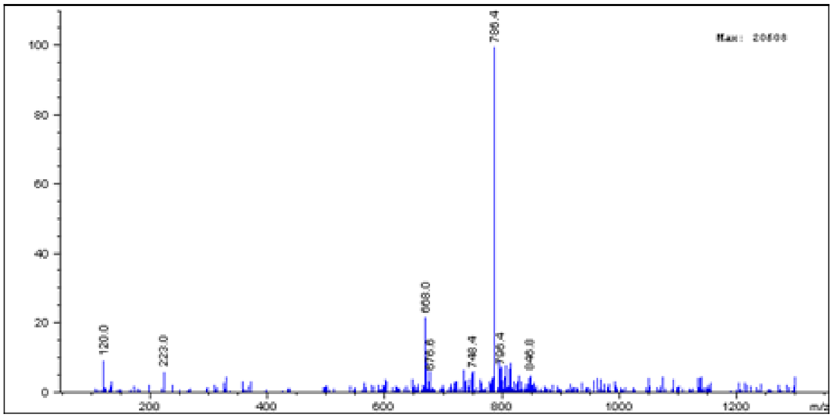

Figure S6. KWCECEFFEFFG peptide ESI-MS results.

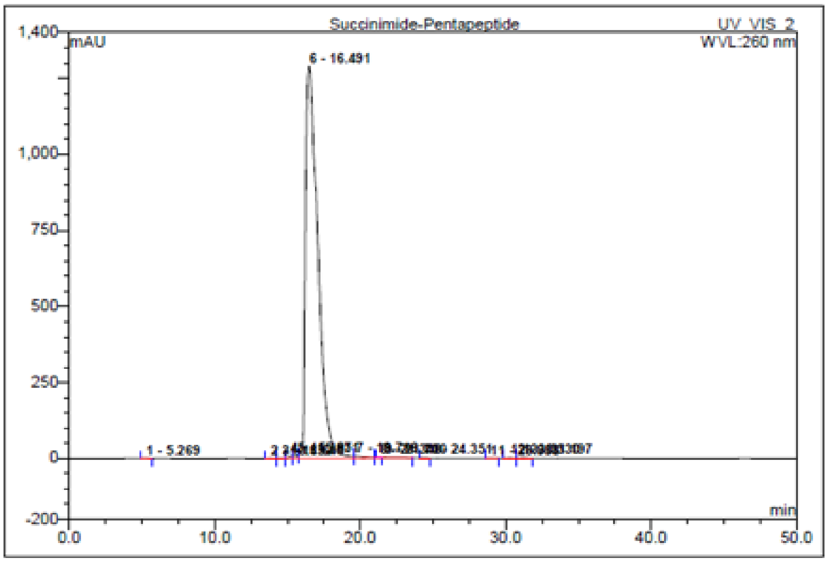

No.	Ret.Time min	Peak Name	Height mAU	Area mAU*min	Rel.Area %	Amount	Type
1	5.27	n.a.	0.602	0.226	0.02	n.a.	BMB
2	13.85	n.a.	0.880	0.391	0.03	n.a.	BM
3	14.65	n.a.	1.437	0.513	0.04	n.a.	M
4	15.20	n.a.	7.527	2.558	0.20	n.a.	M
5	15.67	n.a.	9.258	2.384	0.18	n.a.	M
6	16.49	n.a.	1289.469	1278.076	98.76	n.a.	M
7	19.72	n.a.	6.412	5.147	0.40	n.a.	M
8	21.35	n.a.	0.197	0.050	0.00	n.a.	Ru
9	21.71	n.a.	2.249	3.500	0.27	n.a.	MB
10	24.35	n.a.	0.451	0.161	0.01	n.a.	BMB
11	28.94	n.a.	0.446	0.190	0.01	n.a.	BMB

Figure S7. Succinamic KWCEC peptide HPLC results (98.76% purity).

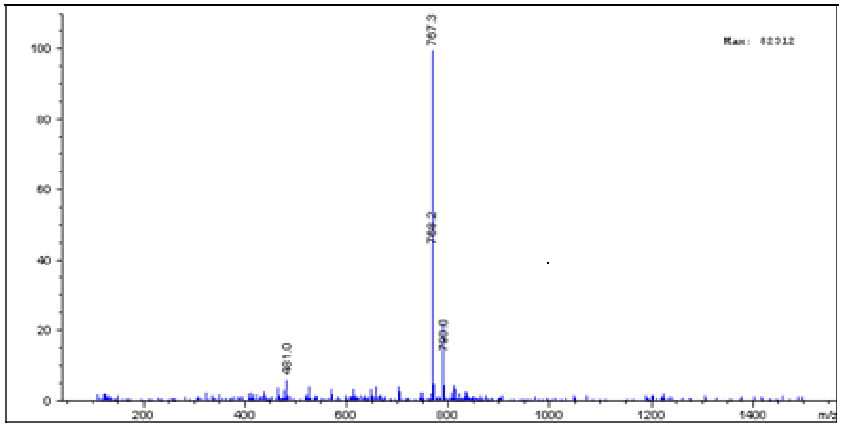

Figure S8. Succinamic KWCEC peptide ESI-MS results.
